# 25 years on and no end in sight: a perspective on the role of RecG protein

**DOI:** 10.1007/s00294-016-0589-z

**Published:** 2016-04-02

**Authors:** Robert G. Lloyd, Christian J. Rudolph

**Affiliations:** 1 Centre for Genetics and Genomics, University of Nottingham, Queen’s Medical Centre, Nottingham, NG7 2UH UK; 2 Division of Biosciences, College of Health and Life Sciences, Brunel University London, Uxbridge, UB8 3PH UK

**Keywords:** Stalled replication forks, Homologous recombination, Stable DNA replication, SDR, Replication termination, Replication fork collisions

## Abstract

The RecG protein of *Escherichia coli* is a double-stranded DNA translocase that unwinds a variety of branched substrates in vitro. Although initially associated with homologous recombination and DNA repair, studies of cells lacking RecG over the past 25 years have led to the suggestion that the protein might be multi-functional and associated with a number of additional cellular processes, including initiation of origin-independent DNA replication, the rescue of stalled or damaged replication forks, replication restart, stationary phase or stress-induced ‘adaptive’ mutations and most recently, naïve adaptation in CRISPR-Cas immunity. Here we discuss the possibility that many of the phenotypes of *recG* mutant cells that have led to this conclusion may stem from a single defect, namely the failure to prevent re-replication of the chromosome. We also present data indicating that this failure does indeed contribute substantially to the much-reduced recovery of recombinants in conjugational crosses with strains lacking both RecG and the RuvABC Holliday junction resolvase.

**Electronic supplementary material:**

The online version of this article (doi:10.1007/s00294-016-0589-z) contains supplementary material, which is available to authorized users.

## Introduction

RecG is a monomeric double-stranded DNA translocase that unwinds a variety of branched DNA molecules in vitro, including Holliday junctions, D-loops, R-loops and various models of replication forks (Lloyd and Sharples 1993; Whitby et al. 1993; Vincent et al. 1996; Fukuoh et al. 1997; McGlynn et al. 1997; Whitby and Lloyd 1998; McGlynn and Lloyd 2000; Briggs et al. 2004; Rudolph et al. 2010b; Manosas et al. 2013; Gupta et al. 2014; Bianco 2015). The protein was first described in *Escherichia coli*, but is present in almost all sequenced species of bacteria (Sharples et al. 1999; Rocha et al. 2005). It is also present in plants where it is targeted to mitochondria and chloroplasts (Odahara et al. 2015; Wallet et al. 2015). However, there appears to be no homologue in fungi or in any animal species.

The *recG* gene was initially identified during a screen for recombination deficient mutants of *E. coli* K12 (Storm et al. 1971), but was not studied in any detail until a further mutation was identified at this locus some 20 years later (Mahdi and Lloyd 1989). A systematic analysis confirmed that inactivation of RecG reduces the recovery of recombinants in conjugational (Hfr × F^−^) crosses. It also confers sensitivity to the crosslinking agent mitomycin C, and a mild sensitivity to UV as well as ionising radiation (Lloyd and Buckman 1991; Lloyd 1991). The reduction in the recovery of recombinants was no more than 2- to 3-fold, a value in sharp contrast to the 100-fold or more reduction seen with mutations that inactivate the RecBCD or RecA recombinases. However, it was in line with the value for strains lacking the RuvABC Holliday junction resolvase (Lloyd et al. 1984). Strikingly, loss of RecG in cells already lacking RuvABC resulted in a much more dramatic reduction, and extreme sensitivity to UV radiation (Lloyd 1991). This strong synergism was interpreted at the time as evidence indicating that RecG and RuvABC might provide partially overlapping pathways for processing intermediates in recombination and DNA repair (Lloyd 1991). However, subsequent studies revealed that the absence of RecG has other effects on the macromolecular metabolism of DNA that might provide for alternative explanations (Rudolph et al. 2010b; Bianco 2015)

We shall return later to consider whether or not RecG does indeed promote recombination. First, we consider what other roles the protein might have. RecG belongs to the Superfamily 2 (SF2) of DNA and RNA helicases (Gorbalenya and Koonin 1993), a family of proteins well known for having multiple functions.

## Limiting pathological events when replication forks meet

Replication of the circular *E. coli* chromosome initiates at a single, sharply defined origin of replication, *oriC*. Two replication fork complexes (replisomes) are established and move away in opposite directions until they meet in a more broadly defined termination area flanked by polar *ter*/Tus fork traps that ensure neither fork is able to proceed beyond this area. Recent marker frequency analyses of logarithmically growing *recG* cells revealed significant over-replication of DNA in this terminus area (Rudolph et al. 2013; Wendel et al. 2014; Dimude et al. 2015). Linearisation of the chromosome eliminates most of this over-replication, consistent with the idea that it stems largely from pathological events associated with the head-on fusion of fork complexes (Rudolph et al. 2013; Dimude et al. 2015).

**Fig. 1:**
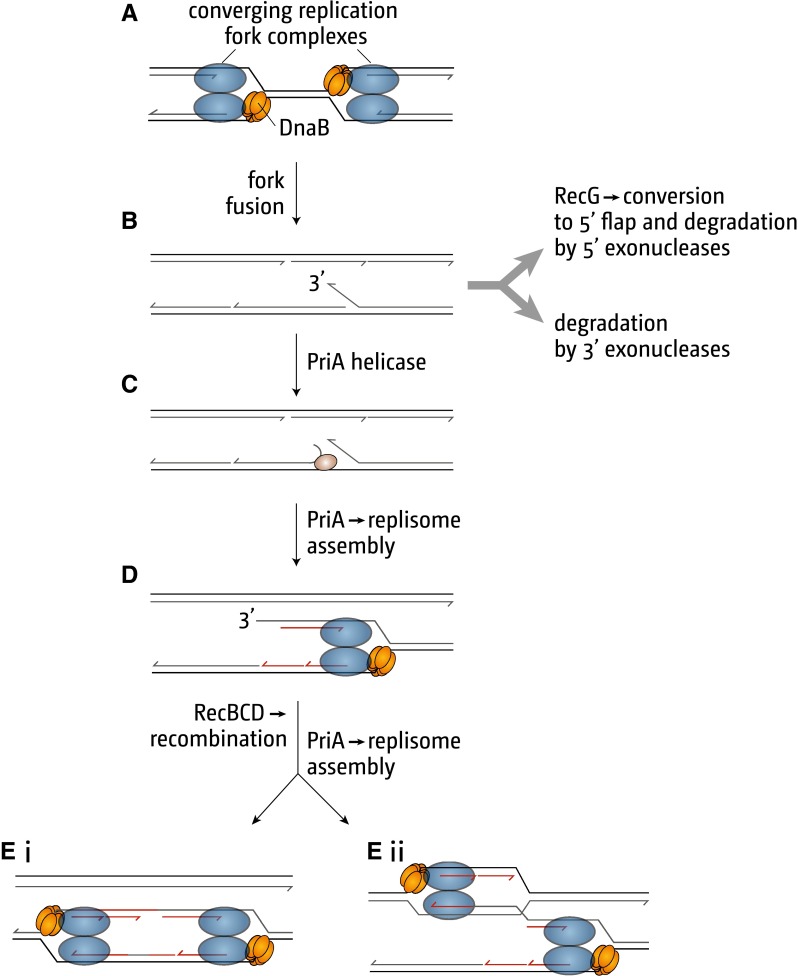
Schematic illustrating how replication fork fusions might lead to the formation of new divergent forks via PriA-mediated replisome assembly and RecBCD-mediated recombination, and how this can be normally suppressed by RecG and/or 3′ exonucleases. The formation of a 3′ flap can occur at both forks. However, for simplicity the schematic details only one such reaction. See text for further details

What happens when replication forks converge is poorly understood, but analysis of the over-replication seen in the absence of RecG indicates that it may result in the production of 3′ single-strand flaps, structures that could be targeted by the primosome assembly protein, PriA, triggering re-replication of the already replicated DNA (Fig. 1a–c) (Rudolph et al. 2010b, 2013). It seems likely that 3′ flaps might either be eliminated by 3′ ssDNA exonucleases, or unwound by RecG and converted to 5′ flaps that could subsequently be removed by 5′ ssDNA exonucleases. This possibility is supported by the observation that eliminating 3′ exonucleases also results in over-replication in the terminus area (Rudolph et al. 2010a, 2013), and the fact that RecG has a particularly high affinity for a 3′ flap (McGlynn et al. 2001; Tanaka and Masai 2006; Bianco 2015). It is also consistent with the inviability of *recG* cells lacking three exonucleases each capable of removing a 3′ flap, and the restoration of viability when the helicase activity of PriA required for the observed over-replication is eliminated (Rudolph et al. 2010a, 2013).

Replication from a 3′ flap would convert the flap to a DNA duplex that RecBCD enzyme might exploit to load RecA (Kowalczykowski 2000), thus provoking recombination and providing PriA with an opportunity to establish another fork that proceeds in the opposite direction (Fig. 1c–e). The two diverging forks would be blocked by the *ter*/Tus traps as they proceed towards *oriC*, explaining why over-replication is tightly restricted to the termination area. Over-replication extends beyond the termination area in both directions if Tus is eliminated. However, it cannot maintain viability without DnaA present to trigger initiation of replication at *oriC* (Rudolph et al. 2013). But it can do so if the strain carries, in addition, a mutation (rpoB*35) that destabilises ternary RNA polymerase complexes (Trautinger et al. 2005; Rudolph et al. 2013). Presumably, replication forks moving out of the termination area would suffer head-on collisions with transcribing RNA polymerase complexes, events normally limited by the replichore arrangement of the chromosome. This ensures that transcription of most highly expressed genes is co-directional with replication (Reyes-Lamothe et al. 2012; Merrikh et al. 2012; Rudolph et al. 2013; Dimude et al. 2015). By reducing conflicts, rpoB*35 enables the forks emerging from the terminus area in *recG tus* cells to replicate the entire chromosome in the absence of origin firing, allowing the cells to grow and divide, albeit with a reduced efficiency (Rudolph et al. 2013).

In a recent study by David Leach and colleagues it was suggested that the over-replication of DNA seen *recG* cells is triggered by fork blockage at *ter*/Tus traps rather than by forks meeting in the terminus area. They proposed that this leads to inappropriate binding of PriA helicase and the establishment of a new fork that replicates back into the terminus area, triggering a pathological cascade (Azeroglu et al. 2016). However, this explanation does not sit well with the fact that over-replication is just as prevalent when fork traps are inactivated (Rudolph et al. 2013).

## Initiating replication at R-loops

Kogoma and co-workers discovered that inactivation of RNase HI in *E. coli* enables chromosome replication and cell viability to be maintained in the absence of initiation at *oriC* (Kogoma 1997). Because RNase HI removes RNA from DNA:RNA hybrids (Horiuchi et al. 1984; Tadokoro and Kanaya 2009), it was suggested that this so-called stable DNA replication (SDR) might initiate at R-loops and they identified particular regions of the chromosome where such initiations are common (de Massy et al. 1984; Kogoma 1997). More recent marker frequency analyses revealed that SDR initiates at a small number of reasonably well-defined chromosomal locations, including a cluster in the termination area (Maduike et al. 2014; Dimude et al. 2015). Because *recG* cells also exhibit elevated SDR (Hong et al. 1995), and RecG protein unwinds RNA from R-loops in vitro (Vincent et al. 1996; Fukuoh et al. 1997), Kogoma and colleagues suggested that the SDR observed in this case might also initiate at R-loops (Kogoma 1997). Indeed, a common basis for initiation might account for the fact that cells lacking RecG and RNase HI both show a peak of synthesis in the termination area of the chromosome (Gowrishankar 2015)

However, this does not appear to be the case. There is no indication that the targeting of 3′ flaps by PriA is required to activate SDR in cells lacking RNase HI, and whereas expression of yeast RNase H1 suppresses this SDR, it has hardly any effect on the SDR detected in cells lacking RecG (Dimude et al. 2015). These findings do not exclude the possibility that RecG dissociates R-loops in vivo, but if it does the absence of this activity may contribute little to the SDR observed in *recG* cells (Dimude et al. 2015), which appears to stem almost exclusively from pathological events initiated in the terminus area of the chromosome.

## Re-starting replication at stalled or damaged forks

The polar *ter*/Tus traps flanking the terminus area of the *E. coli* chromosome dictate that the two replication forks established at *oriC* must both reach the terminus area in order for chromosome duplication to be completed (Reyes-Lamothe et al. 2012). Any block to the progression of either fork is therefore a potential threat to survival (McGlynn and Lloyd 2002; Syeda et al. 2014). The increased sensitivity of *recG* cells to killing by agents that damage DNA (Mahdi and Lloyd 1989), and especially the synergy observed when *recG* is combined with other mutations that compromise survival (Lloyd 1991; Cooper et al. 2015) indicates that RecG has a significant role to play in securing duplication of the chromosome when the DNA is damaged. But in what capacity?

**Fig. 2:**
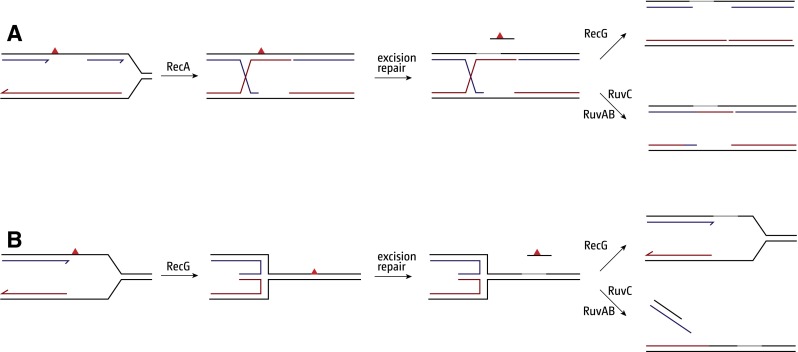
Models for damage avoidance pathways triggered by UV-induced lesions that interfere with DNA replication. **a** Replication skips the lesion (*red triangle*), leaving a single-stranded gap that needs filling by homologous recombination. **b** If the replication fork stalls at a UV-induced lesion, replication fork regression can transfer the lesion back into double-stranded DNA for repair via excision repair. See text for details

Clues as to how chromosome replication is completed came from early studies of how cells survive exposure to UV radiation. UV light introduces pyrimidine dimers into DNA, lesions that block polymerisation by replicative polymerases. Rupp and Howard-Flanders suggested that forks simply skip over each dimer, leaving single-strand gaps in the daughter duplexes to be repaired subsequently via homologous recombination (Rupp and Howard-Flanders 1968). Strand exchange between the gapped daughter and its intact sister mediated by the RecA recombinase places the pyrimidine dimer in duplex DNA once more, enabling its removal by UvrABC-mediated excision repair system (Fig. 2a) (West et al. 1981). However, the completion of repair would require further processing of the strand exchange intermediate, coupled with new DNA synthesis to close any remaining gap. RuvABC-mediated resolution of any Holliday junction established, or reversal of the initial strand exchange via branch migration mediated by RecG or RuvAB, would dissociate the physical link between the two sisters (Fig. 2a) (Whitby et al. 1993). Either mechanism would be consistent with the synergistic effect of both *ruv* and *recG* mutations on the UV-sensitivity of excision-defective *uvr* mutants (Lloyd et al. 1984; Lloyd and Buckman 1991). The absence of both mechanisms might be sufficient to account for the extreme sensitivity of *ruv recG* strains (Lloyd 1991).

The discontinuous nature of lagging strand synthesis made it easy to accept that the lagging strand polymerase would be able to resume synthesis downstream of a lesion. But synthesis of the leading strand was thought to be continuous and it was suggested that in this case a lesion in the template might bring fork progression to a halt (Meneghini and Hanawalt 1975). However, Marians and co-workers have now demonstrated that the replisome complex can re-prime synthesis downstream of a lesion blocking synthesis by the leading strand polymerase, at least in vitro, thus potentially overturning what was considered to be a significant objection to the Rupp and Howard-Flanders model (Heller and Marians 2005, 2006a, b).

But the fact remains that DNA synthesis ceases abruptly following irradiation with a moderate dose of UV light, but normally resumes after a short delay (Hanawalt and Setlow 1960), an observation made repeatedly over subsequent years (Khidhir et al. 1985; Courcelle et al. 2005, 2006; Rudolph et al. 2007). The cessation of synthesis observed in vivo is consistent with the observation that skipping lesions in the leading strand template in vitro is not 100 % efficient. Thus fork stalling is likely to occur in vivo when there are multiple lesions in the DNA (Yeeles and Marians 2011). With the DNA unwound, and the fork stalled, it seemed unlikely that the lesion in the leading strand template could be removed by the nucleotide excision repair system. So how might replication resume?

Transient replacement of the replicative polymerase with an alternative polymerase that has the ability to extend the nascent strand across the site of damage would be one simple solution (Goodman and Woodgate 2013; Fuchs and Fujii 2013). However, such translesion synthesis (TLS) comes at the cost of being error-prone (Goodman and Woodgate 2013). Recent studies have also indicated that TLS and lesion skipping are competing activities for resuming synthesis (Gabbai et al. 2014). Which would normally predominate is not clear.

A quite different solution emerged from an earlier observation indicating that blocked forks can reverse to form a Holliday junction or »chicken foot« structure (Higgins et al. 1976; Fujiwara and Tatsumi 1976). Fork reversal would return the lesion to a region of double-stranded DNA, enabling its excision (Fig. 2b), while exonuclease-mediated digestion of the extruded nascent strands, or branch migration of the junction in the opposite direction, would re-establish a fork that PriA might then target to assemble a new replisome complex, enabling replication to restart in an error-free manner (McGlynn and Lloyd 2000, 2002; McGlynn et al. 2001; Manosas et al. 2013; Gupta et al. 2014). Indeed, it seems that an error-free lesion bypass mechanisms might be preferred over error-prone TLS (Courcelle et al. 2006).

But what might drive fork reversal and how might replication resume? A possible scenario emerged when *E. coli* RecG protein was shown to catalyse the interconversion of fork and Holliday junction structures in vitro (McGlynn and Lloyd 2000), a result recapitulated with RecG proteins from other bacteria (Zegeye et al. 2012; Thakur et al. 2015). Indeed, recent in vitro studies exploiting substrates and conditions that more closely mimic the situation likely to be encountered at a stalled fork in vivo confirmed that RecG is able to drive fork reversal and revealed that dissociation of the replisome is probably not a pre-requisite (Gupta et al. 2014). They also revealed that RecG stimulates fork reversal mediated via the branch migration activity of RuvAB, consistent with a previous suggestion that the likely starting material for RuvAB is a Holliday junction (McGlynn and Lloyd 2001).

The idea that RecG might reverse forks stalled at DNA lesions and thereby enable replication to restart via the formation and subsequent processing of a Holliday junction intermediate was particularly tempting in light of the established genetic interaction between RecG and the restart protein PriA (Al-Deib et al. 1996; Gregg et al. 2002; Jaktaji and Lloyd 2003; Zhang et al. 2010). It also provided an appealing connection between recombination and replication.

However, evidence that RecG reverses stalled replication forks in vivo is conspicuous by its absence. There is no indication that RecG contributes significantly to the replication fork reversal detected when forks have been stalled through direct inactivation of replisome components (Seigneur et al. 2000; Michel and Leach 2012). Instead, fork reversal in these situations seems to be catalysed by RuvAB, and indeed *ruvA* mutations have been identified that interfere specifically with this reaction (Baharoglu et al. 2008; Le Masson et al. 2008). Perhaps RecG might be brought into play only when the DNA itself is damaged. However, Courcelle and co-workers found no evidence to suggest that the recovery of DNA synthesis in UV-irradiated cells is significantly delayed in the absence of RecG (Donaldson et al. 2004). In addition, *recG* cells show a mildly reduced spontaneous mutation rate to rifampicin resistance (Table S1), despite the fact that the SOS response is constitutively increased in *recG* cells (O’Reilly and Kreuzer 2004), indicating that TLS is unlikely to be compensating for the absence of RecG, enabling replication to resume at about the same time as in the wild-type.

So, is there still reason to suspect that RecG is actively involved in rescuing stalled forks? Some might say no, but a final verdict will probably have to wait until methods become available to more directly visualise what happens at individual stalled forks in vivo. What cannot be disputed is that the absence of RecG has a profound effect on the recovery of normal chromosome replication and cell division following irradiation with UV light (Rudolph et al. 2009a).

## Chromosome replication and segregation

**Fig. 3:**
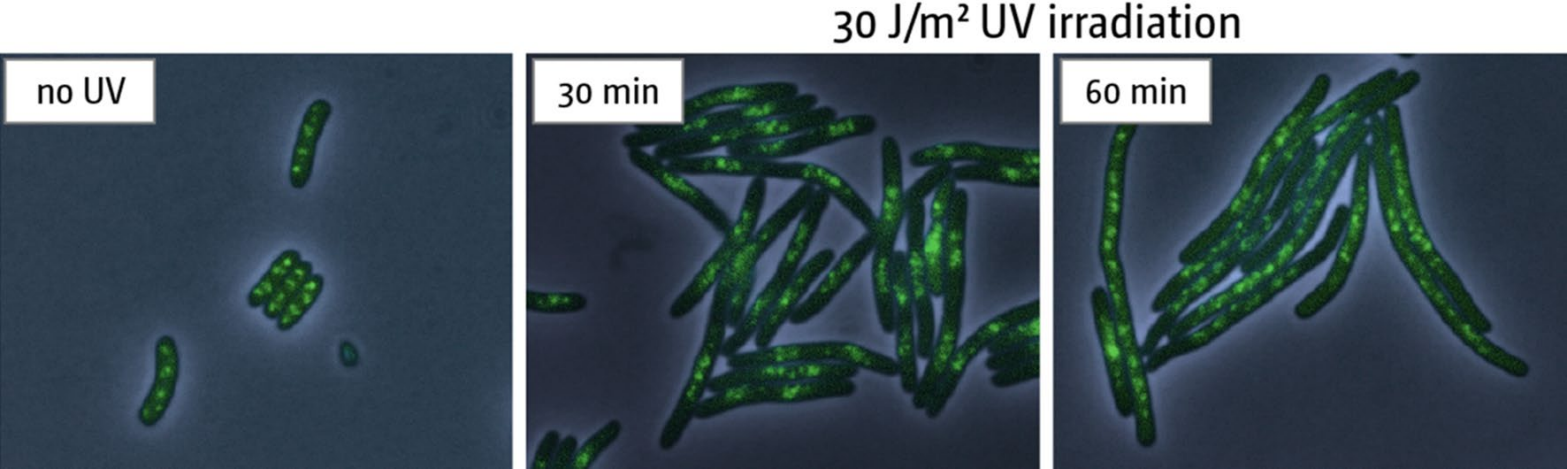
Effect of UV-induced damage on the number of replisomes in wild type cells. Replisomes are visualised by fusing YPet to the sliding clamp DnaN (AS1062, MG1655 YPet-DnaN; see Table S4). Cells were irradiated with 30 J/m^2^ UV and incubated under agitation. Samples were taken at the times indicated. Combined phase contrast and fluorescence images are shown

Analysing what happens to DNA replication in cells that have been irradiated with UV light is complicated by the fact that at least three processes contribute to the net DNA synthesis detected subsequently: (a) the restart of replication at stalled forks, (b) continued firing of *oriC* at regular intervals and (c) the establishment of new forks at sites other than the origin (Rudolph et al. 2007). The last of these was initially reported by Kogoma et al. and referred to as inducible stable DNA replication (iSDR) (Kogoma 1997). As might be expected from this unscheduled initiation, irradiated wild-type cells show a substantial increase in the number of replication fork complexes (Fig. 3).

The temporary cessation of DNA synthesis immediately after irradiation provides a window of opportunity for excision repair to clear the way ahead for any fork that has been rescued in the interim (Donaldson et al. 2004; Rudolph et al. 2007, 2008). But until then new forks established at *oriC* accumulate in wild-type cells, amplifying the origin region (Rudolph et al. 2007). This coincides with a period of cell filamentation that lasts for some 60 min following irradiation during which there is an accumulation of replisomes (Fig. 3) and ultimately of fully replicated chromosomes, but no increase in the number of viable cells. It is followed by a period of rapid cell division with a reduced generation time of ~15 min, somewhat reducing the delay caused by filamentation. Following these rapid divisions, growth returns to the same rate as in unirradiated cells (Rudolph et al. 2007, 2009b, 2010b).

**Fig. 4:**
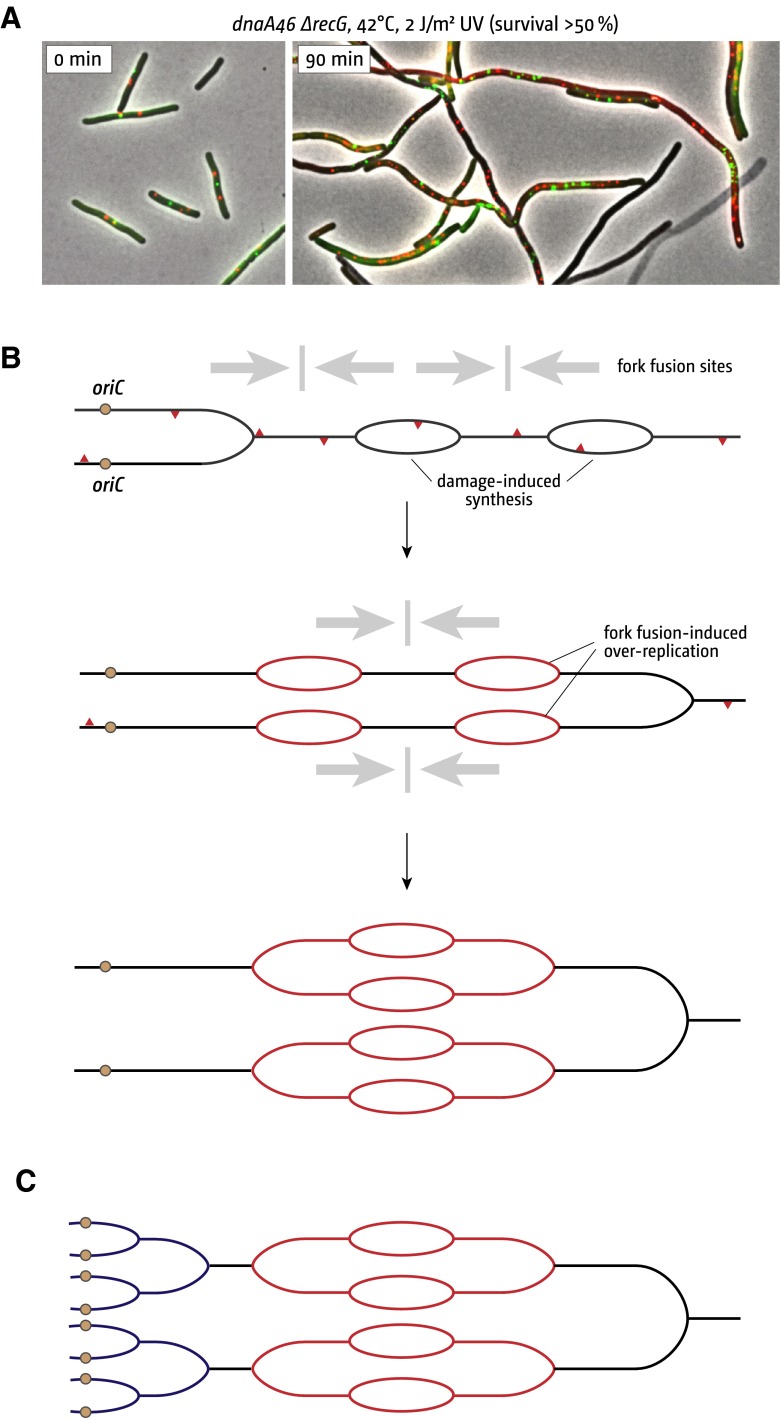
An increased number of fork fusion events caused by UV-induced origin-independent synthesis leads to uncontrolled DNA amplification. **a** UV-irradiation leads to a drastic and often unbalanced increase in origin (*red*) and terminus (*green*) foci in the absence of *oriC* firing (combined phase contrast and fluorescence images are shown). Cells were grown at permissive temperature prior to UV treatment and shifted to 42 °C directly after irradiation. The strain used was RCe198 (*recG dnaA46*). **b**, **c** Schematic for the pathological cascade triggered by converging replication forks in the absence of RecG. UV-induced damage is indicated by *red triangles*. Over-replication by fork fusion events (positions of fusion events indicated by *grey arrows*) is highlighted in *red*, synthesis from origin firing in *blue*

This sequence of events is clearly disrupted in the absence of RecG. Even when the UV dose results in little loss of viability the cells have great difficulty resuming normal growth and division. Each cell filaments profusely (Ishioka et al. 1997; Rudolph et al. 2009a), and continues doing so until at least one progeny cell capable of normal growth and division emerges from the filamentous mass after a quite a prolonged delay (Ishioka et al. 1997; Rudolph et al. 2009a). Eliminating SulA (SfiA), the SOS-induced inhibitor of cell division, does not suppress this phenotype. There is substantially increased DNA synthesis during this post-irradiation period, both in the presence and absence of origin firing (Donaldson et al. 2004; Rudolph et al. 2009a, b). There is also significant amplification of both origin and terminus areas of the chromosome. However, these areas fail to segregate properly as they do in wild-type cells, and instead form loosely associated clusters (Rudolph et al. 2009a). Similar experiments in *dnaA recG* cells irradiated with doses allowing more than 50 % of cells to survive revealed that even in the absence of origin firing there is a dramatic and often unbalanced amplification of limited areas of the chromosome (Fig. 4a) (Rudolph et al. 2009a, b).

Rather than reflecting a failure to rescue stalled forks, might this phenotype instead be a consequence of the pathological replication triggered via the action of PriA and exacerbated by initiation of iSDR? The extra forks established in the irradiated cells would further disrupt the replichore arrangement of the chromosome, leading to unscheduled amplification of the replicated areas and increasing the incidence of fork fusions that might trigger further over-replication of the DNA (Fig. 4b, c). It would also increase conflicts with transcription, which itself is likely to have pathological consequences, especially at highly transcribed genes such as *rrn* operons (Trautinger et al. 2005; Wang et al. 2007; Guy et al. 2009; Boubakri et al. 2010; Srivatsan et al. 2010; Atkinson et al. 2011; De Septenville et al. 2012; Merrikh et al. 2012; Dimude et al. 2015; Ivanova et al. 2015). Recombination initiated via RecBCD- and RecFOR-mediated loading of RecA might further compound the problem by linking chromosomes and/or partially replicated areas together. This scenario certainly fits with the fact that eliminating the helicase activity of PriA needed to initiate over-replication enables irradiated *recG* cells to recover much more quickly (Rudolph et al. 2009b). It is also consistent with the requirement for RuvABC resolvase to maintain the viability of *recG* cells carrying additional mutations that enable them to grow and divide without origin firing (recG dnaA tus rpo*35 cells) (Rudolph et al. 2013).

So, rather than specifically promoting recombination and allied aspects of DNA replication and repair, RecG might instead be a factor that simply prevents the escalation of pathological events when DNA is damaged. However, we cannot exclude the possibility that it might act in both capacities.

## Homologous recombination

Might the over-replication of DNA in cells lacking RecG, or more specifically any recombination triggered by such pathological replication, account for the much-reduced recovery of recombinants in Hfr crosses with *ruv recG* double mutants? Several studies have shown that mutations known to trigger hyper-recombination (*dam, polA, uvrD*) are synthetically lethal with *ruv* (Florés et al. 2005; Magner et al. 2007; Lestini and Michel 2007; Zhang et al. 2010). Robust viability is restored by expressing the RusA Holliday junction resolvase (Sharples et al. 1994), confirming that the failure to process junctions via a canonical resolvase is the reason for the inviability rather than any lack of RuvAB-mediated branch migration (Zhang et al. 2010).

**Fig. 5:**
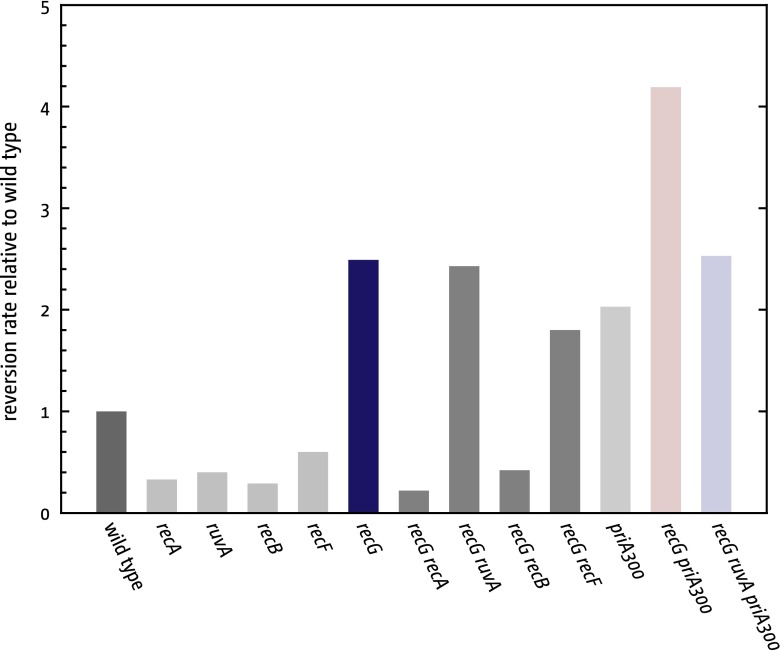
Effect of recombination deficiencies on spontaneous rates of tandem repeat recombination events. The recombination reporter plasmid containing the tandem repeat cassette called KanKanMX4 (Ede et al. 2011) contains an internal 266 bp duplication which leads to inactivation of the kanamycin resistance gene. Loss of the repeated sequence is scored by restoration of kanamycin resistance. The spontaneous reversion rates were measured as described (see Supplementary Methods). Spontaneous rates are given relative to the respective spontaneous rate in wild type cells, which was set to 1

The absence of RecG is known to increase certain types of recombination. For instance, Lovett et al. reported that the frequency of deletions between tandem repeat sequences located either on a plasmid or on the chromosome is increased in *recG* cells but reduced in *ruv* cells (Lovett et al. 1993; Lovett 2006). We recapitulated these findings using a plasmid-based assay (Fig. 5; Table S2). We also found that the increase seen with *recG* cells depends on the function of both RecA and RecBCD but not that of RuvABC (Fig. 5). Thus, it appears the absence of RecG can indeed provoke increased recombination, and via a mechanism that exposes a target for RecBCD enzyme. Thus, the presence of RecG may limit certain types of genetic exchanges. However, since *ruv recG* cells are viable, the absence of RecG clearly is not particularly detrimental to survival during normal growth, though it should be noted that viability is reduced by some twofold more than in a *ruv* single mutant (Lloyd 1991) (Table S3).

But might that situation change in conjugational crosses with Hfr donors? Because of the linear nature of the DNA fragment transferred from the Hfr, at least two exchanges are believed to be required in order to produce a viable (circular) product. As discussed below, at least one of these exchanges might be expected to establish a replication fork, adding to those already traversing the recipient chromosome, compromising the normal replichore arrangement. If in the absence of RecG this were to set in motion a cascade of pathological replication that provoked recombination, the viability of the zygotic cells would be compromised, especially if RuvABC was also missing. It might account for the observed reduction in the recovery of haploid recombinants (Lloyd 1991). Under the experimental conditions used (a tenfold excess of recipient over Hfr cells) the loss of viable cells from the recipient population would be easily missed.

We investigated this possibility by introducing into a *ruv recG* recipient a mutation (*priA300*) that eliminates the helicase activity of PriA and which we knew to prevent over-replication of the terminus area in *recG* cells (Rudolph et al. 2013). We found that its presence makes hardly any difference to the recovery of recombinants (Table 1). This was unexpected given that *priA300* is otherwise a strong suppressor of the *recG* mutant phenotype (Al-Deib et al. 1996; Gregg et al. 2002; Jaktaji and Lloyd 2003; Rudolph et al. 2009a, 2013; Zhang et al. 2010; Mahdi et al. 2012). The presence of *priA300* alone has no substantial effect on the numbers of recombinants recovered (Table 1; Table S3). However, it increases recombination between plasmid-encoded tandem repeats and accentuates the increase seen in the absence of RecG in a manner that depends on the function of RuvABC (Fig. 5). This, together with the ~4-fold reduction in the recovery of recombinants seen in conjugational crosses with a *priA300 ruv* strain (Table 1) and the increased sensitivity of the double mutant to killing by UV light (Jaktaji and Lloyd 2003) indicates that the absence of PriA helicase activity may itself result in a general increase in recombination events that require resolution of Holliday junctions for their completion.

**Table 1:** Efficiency of homologous recombination in Hfr × F^−^ crosses

F^−^ recipient cell genotype	Effect of additional recipient mutations on the efficiency of recombination			
	None	*priA300*	*recF*	*priA300 recF*
*Wt*	1.00	0.82 ± 0.09	0.71 ± 0.02	0.70 ± 0.1
*recG*	0.46 ± 0.09	0.90 ± 0.06	0.3^a^	0.60 ± 0.07
*ruvABC*	0.72 ± 0.18	0.22 ± 0.02	1.35 ± 0.28	0.97 ± 0.05
*ruvABC recG*	0.0061 ± 0.0001	0.0077 ± 0.0026	0.044 ± 0.005	0.25 ± 0.03
*ruvABC recG recO recB*		0.00095 ± 00018		

Values are from crosses with Hfr donor strain GY2200, unless specified, and are means of at least two independent experiments, usually three or more, corrected for any reduction in the viability of the recipient cells. Error estimates are standard errors of the mean values. Uncorrected mean values, measures of recipient cell viability and of mating efficiency are documented in Table S2 along with the number of experiments conducted with each recipient

^a^Data reproduced from (Ryder et al. 1994)

A previous study of conjugational recombination in *ruv recG* cells revealed that inactivation of any one component of the RecFOR complex improves the recovery of recombinants by about sevenfold (Ryder et al. 1994). When we tested a *recF* null allele in combination with *priA300* we observed a 40-fold increase (Table 1). This remarkable effect eliminates virtually all of the synergism between the *ruv* and *recG* mutations. Combining *priA300* with mutations inactivating *recO*, *recR*, *recQ* or *recJ* has the same effect, except when in the case of *recR*, early transfer of the wild-type allele by the particular Hfr strains used compromises the recovery (Table S3). Significantly, recovery of recombinant progeny requires the presence of functional RecBCD enzyme (Table 1, Table S3) (Ryder et al. 1994).

So, it would appear that in terms of conjugational recombination, and in the absence of RuvABC, the critical role of RecG is perhaps not so much to provide an alternative route for the processing of Holliday junctions as (a) to curtail some negative effect of PriA helicase activity, a role initially proposed by Al-Deib et al. (1996) and recently re-visited by Azeroglu et al. (2016), and (b) to prevent RecFOR from loading RecA. How might RecG achieve both?

It has been suggested that the presence of RecG might stabilise D-loop intermediates formed during RecBCD- and RecA-mediated recombination. Whitby et al. (1995) proposed that by driving branch migration away from the 3′ end of the invading strand RecG might help to establish a Holliday junction that could then be resolved by RuvABC, enabling PriA to convert the D-loop to a replication fork. Recently, Azeroglu et al. (2016) proposed instead that RecG is needed to prevent PriA helicase from loading at a D-loop in such a way as to unwind and dissociate the structure. Both models are consistent with the fact that overexpressing PriA has a strong negative effect on the efficiency of recombination and DNA repair in *recG* mutant cells (Al-Deib et al. 1996). Both suggestions also fit with the fact that mutations encoding helicase defective PriA proteins suppress most features of the *recG* mutant phenotype (Al-Deib et al. 1996). However, there is one significant exception. These *priA* alleles do not eliminate the strong synergism between *ruv* and *recG* mutations, neither with respect to recombination nor DNA repair (Table 1) (Al-Deib et al. 1996; Jaktaji and Lloyd 2003). But they can do so for conjugational recombination provided RecFOR is inactivated (Table 1, Table S2). This conditional suppression is not easily reconciled with the idea that RecG is required simply to stabilise D-loops. It may well do so, but is not enough to allow efficient recovery of recombinants. Inactivation of RecFOR only marginally improves the survival of UV-irradiated *ruv recG priA300* cells (data not shown), but this is not surprising as RecFOR is required for post-replication recombination repair (Rupp and Howard-Flanders 1968; Heller and Marians 2006a). What might the RecFOR complex be doing that prevents *priA300* from suppressing the synergism between *ruv* and *recG*?

During conjugation between Hfr donors and F^−^ recipient, a single-strand of Hfr DNA is transferred to the recipient cell with a 5′–3′ polarity where it provides a template for lagging strand synthesis (Willetts and Wilkins 1984). While transfer is in progress, the leading 5′ end is probably attached to DNA helicase I at the site of transfer replication so that in effect a growing loop of partially duplex DNA is presented to the recipient (Matson et al. 1993). When mating is interrupted, deliberately or spontaneously, the recipient is left with a linear fragment of Hfr DNA that may not be a full duplex, at least initially, and which has ~40 kb of F-plasmid DNA at the leading end and a 3′ ssDNA overhang at the distal end due to the polarity of lagging strand synthesis (Lloyd and Buckman 1995). Homologous pairing and strand exchange between the donor and recipient DNA mediated by RecA recombinase leads to the formation of haploid recombinants. An even number of exchanges is assumed to be required to maintain a circular chromosome (Smith 1991).

Genetic analyses indicate that the majority of the recombinants arise from just two exchanges (Lloyd and Buckman 1995). Given that the recombinase activity of the RecBCD enzyme complex is essential, it is tempting to believe that both exchanges initiate at or near the ends of the transferred DNA. RecBCD has been shown to unwind and degrade DNA from a duplex or near duplex DNA and after an encounter with a χ DNA sequence to expose a single-strand ending 3′ on which it then loads RecA, establishing a RecA-nucleoprotein filament that initiates homologous pairing and strand exchange, and a D-loop that PriA may then exploit to build a replication fork (Kowalczykowski 2000; Wigley 2013).

However, RecA can also be loaded on gapped DNA via the combined activities of the RecF, RecO and RecR proteins (Morimatsu and Kowalczykowski 2003; Morimatsu et al. 2012). Indeed, the spectrum of recombinant genotypes observed in Hfr crosses is consistent with one exchange being initiated via RecFOR-mediated loading of RecA at a transient single-strand gap in the transferred Hfr DNA while transfer is in progress, and the second via RecBCD-mediated loading of RecA at a DNA end once transfer had terminated, most likely at the distal end (Lloyd and Buckman 1995). When the RecFOR system is inactivated, recombinants arise efficiently from two RecBCD-mediated exchanges, one initiated near each end of the transferred Hfr DNA. Assuming both exchanges establish replication forks, divergent replication towards the terminus might provide a mechanism for producing haploid recombinant progeny (Smith 1991), though one of the two forks would contravene the replichore arrangement for some of its journey, which might cause problems. The requirement for at least one RecBCD-mediated initiation event accounts for the 100-fold or more reduction in the recovery of recombinants in crosses with *recB* and *recC* mutant recipients.

The idea that conjugational recombination might frequently initiate via a RecA-mediated exchange at a single-strand gap came initially from evidence of abortive recombination in *ruv* mutants lacking functional RecBCD enzyme (Benson et al. 1991). A more recent study indicated that such exchanges are particularly frequent when PriB protein is missing, occurring perhaps in ≥99 % of DNA transfer events (Mahdi et al. 2012). They also prevent the recovery of progeny when RuvABC is also missing, unless the RusA resolvase is activated, indicating that exchanges initiated this way lead to the formation of a Holliday junction and that these junctions can be resolved only via the activity of a canonical resolvase. Significantly, the presence of RecG does not appear to help in this circumstance. Inactivating RecFOR restores efficient recovery of progeny, establishing that the exchanges likely initiate at gaps. It was suggested that in a *rec* ^+^ strain the absence of PriB might delay gap closure, increasing the likelihood of RecA loading via RecFOR (Mahdi et al. 2012).

The fact that it is possible for recombination to initiate at a gap with very high frequency provides a new way to explain why the recovery of recombinants in conjugational crosses with *ruv recG* recipients is so low, and how combining *priA300* with inactivation of RecFOR restores recovery close to wild-type levels. We suggest the initial strand exchange initiated via RecFOR is often aborted by driving the branch point in the reverse direction to that catalysed initially by RecA, a reaction that RecG would seem well able to do (Whitby et al. 1993). When the exchange is aborted this way, recombinants arise subsequently via RecBCD-mediated exchanges initiated at the ends of the Hfr fragment released into the recipient. The absence of RecG would stabilise an exchange at a gap, enabling a Holliday junction to be established and covalently sealed in a joint molecule intermediate, an intermediate that we suggest can be resolved only via a canonical resolvase like RuvABC or RusA, as stated above. But the absence of RecG would also lead to over-replication of DNA via the primosome assembly activity of PriA, triggering and stabilising further (RecBCD-mediated) exchanges that would compromise viability in the absence of RuvABC even if the RecFOR system had been inactivated to reduce or eliminate the initiation of recombination at gaps. When the potential for over-replication is curbed by the presence of *priA300*, the full effect of inactivating RecFOR becomes manifest. Without *priA300*, inactivating RecFOR has limited success. Without inactivating RecFOR, *priA300* may prevent pathological replication, but cannot do anything about the Holliday junctions generated as a result of initiating recombination at gaps, thus explaining why a functional RecFOR complex is epistatic over *priA300*.

However, one note of caution is that the presence of RecG is not in itself sufficient to prevent abortive exchanges in crosses with *ruv* *priB* recipient cells (Mahdi et al. 2012), as it might be expected to if it dissociates recombination intermediates generated via RecFOR-mediated loading of RecA. Perhaps the absence of PriB delays the normal closure of these gaps, as suggested (Mahdi et al. 2012), swamping the ability of RecG to cope.

An important corollary to this work is that the RecBCD-dependent system of initiating genetic exchanges remains able to produce recombinant progeny quite efficiently in the absence of a canonical Holliday junction resolvase such as RuvABC, as originally reported (Otsuji et al. 1974; Lloyd et al. 1984). Holliday junctions are either not generated or can be processed to yield viable products by an alternative resolvase, or some other type of nuclease that attacks branched DNAs. Eukaryotes have several nucleases that resolve Holliday junction structures (Sarbajna et al. 2014). The RusA protein of *E. coli* resolves Holliday junctions much like RuvC and can provide an efficient alternative to RuvABC provided RecG is present. However, the *rusA* gene is not normally expressed and its deletion is without detriment to the recovery of recombinants in crosses with *ruv* mutants (Mandal et al. 1993; Mahdi et al. 1996; Zhang et al. 2010). Zhang et al. presented evidence that any other nuclease wild-type *E. coli* may have that could act in concert with RecG to resolve Holliday junctions must operate very inefficiently indeed compared with RusA (Zhang et al. 2010).

Wardrope et al. suggested that Holliday junctions might be removed by driving their branch migration until they merge with replication forks (Wardrope et al. 2009). But would this work in *ruv recG* cells when *priA300*, coupled with the inactivation of RecFOR, enables RecBCD to deliver recombinant progeny with an efficiency equal to 25 % of that in the wild-type?

Studies by Lopez et al. reported that inactivating topoisomerase III reduces the recovery of recombinants in transductional crosses with a *ruvC* mutant and proposed a mechanism by which topoisomerase activity provides an alternative to RuvABC-mediated resolution for the elimination of Holliday junctions (Lopez et al. 2005). However, the observed reduction in recombination was only some twofold, and there were signs in this study that the loss of topoisomerase III is detrimental to the viability of *ruvC* cells. Topoisomerases have multiple, often essential activities in the cell and their depletion can attract compensating suppressors that make it difficult to assess their contribution to a particular cellular activity (Stockum et al. 2012). So, how RecBCD might be able to promote recombination in the absence of a canonical resolvase remains to be established.

## Conclusions

Despite extensive in vivo and in vitro investigations over the last 25 or so years, exactly what RecG does in the cell is still very much a matter of debate. Most of the suggestions that have been made emerged from studies with *recG* strains exposed to agents that damage DNA or which carry one or more additional mutations that compromise DNA macromolecular metabolism, chromosome segregation or cell division, circumstances that could easily cloud the issue. As we have discussed, some studies cast genuine doubt regarding certain proposals made at an earlier stage, but negative findings in other cases may simply reflect limitations of the experimental approaches exploited.

Our discovery that a *recG* single mutant growing exponentially in the absence of any factor that might compromise DNA metabolism displays an over-replication of DNA that is specific to the terminus area of the chromosome is therefore a game-changer. Together with our findings from the analysis of what triggers this replication, it has shed light on an aspect of cell biology that is poorly understood, namely what happens when two converging forks meet to complete replication of the chromosome. This discovery has enabled us to review previous suggestions about the function of RecG, and in particular to inspect closely its contribution to the recovery of recombinants in conjugational crosses. We are tempted to conclude that it contributes very little, and to suggest instead that the poor recovery of recombinants observed in crosses with *ruv recG* strains is a consequence of two events, both resulting from the absence of RecG: (a) an inability to abort exchanges that lead to the formation of Holliday junctions that can be processed further only via the action of RuvABC, and (b) the failure to curb pathological events triggered when converging replication forks meet.

However, the fact that RecG is present in wild type cells, and the protein has an undeniable ability to efficiently interconvert replication fork and Holliday junction structures in vitro means we cannot dismiss the idea that RecG normally contributes to the generation of recombinants in conjugational crosses. Indeed, any role that has been attributed to the protein on the basis of its ability to bind and unwind particular branched DNA substrates in vitro remain a possibility until studies of protein-DNA interactions in vivo have reached such an advanced state of sophistication that it can be rigorously excluded. Until then some proposed roles will have more appeal than others.

## Electronic supplementary material

Below is the link to the electronic supplementary material.

Supplementary material 1 (DOCX 90 kb)
