# Supplementary material for: 25 years on and no end in sight: a perspective on the role of RecG protein
Source: Curr Genet. 2016 Apr 2;62(4):827–40. doi: 10.1007/s00294-016-0589-z (PMC5055574; doi:10.1007/s00294-016-0589-z)
Supplement: Supplementary file 1 — Supplementary material 1 (DOCX 90 kb) [file 294_2016_589_MOESM1_ESM.docx]

# Supplementary Methods

## Growth media

All bacterial strains are listed in Table S4. Strains were constructed via P1*vir* transductions (Thomason et al. 2007) or single-step gene disruptions (Datsenko and Wanner 2000). Luria broth (LB) and minimal medium “56” was prepared as described (Dimude et al. 2015).

## Plasmids used in this study

Plasmid pAM401 was described elsewhere (Rudolph et al. 2010). The pRS316-kankanMX4 plasmid was generated as described (Ede et al. 2011). In brief, an internal 266 bp *Nsi*I fragment of the KanMX4 resistance cassette from plasmid pFA6a-KanMX4 (Wach et al. 1994) was cloned into pFA6a-KanMX4 partially cleaved with *Nsi*I, thereby creating an internal direct 266 bp duplication which leads to inactivation of the resistance gene. The module was subcloned via *Eco*RI and *Sal*I into shuttle vector pRS316 (Sikorski and Hieter 1989), sequenced and subsequently used in the backgrounds indicated.

## Genetic crosses and measures of recombination

F-prime and Hfr donors were mated with F^–^ recipient strains in high-salt LB broth at 37°C as described (Lloyd et al. 1987; Lloyd et al. 1988; Mahdi et al. 2012). Measurements of cell viability relate to the number of cfu in the recipient culture at an *A_650_* of 0.4, as determined with plating on non-selective 56/2 agar. All recipients were derivatives of the multi-auxotrophic, streptomycin-resistant strain, AB1157 (Table S1). Transconjugants were selected using 56/2 or LB agar, as appropriate, supplemented with 100 mg/ml streptomycin to counterselect donor cells. Transductions were conducted using phage P1*vir* transductions (Thomason et al. 2007).

## Spontaneous mutation rates

Spontaneous mutation rates were determined by the method of the median (Lea and Coulson 1949; Foster 2006). Appropriate dilutions from an overnight culture of the strain of interest were spread on LB agar plates to allow single cells to grow into colonies. 9 colonies were completely cut out of the agar with a sterile scalpel blade and transferred into a culture tube with 5 ml LB broth. The resulting 9 tubes were incubated for 24 h with agitation until bacterial growth reached stationary phase. 200 µl aliquots from all cultures were plated onto LB containing 10 mg/ml rifampicin to determine the number of spontaneously resistant mutants in each culture. The viable titre was determined by plating appropriate dilutions of 3 randomly chosen cultures onto LB agar plates. The mean of the viable titres was used in the calculation of the mutation rate. For all experiments the mutation rate of wild type and mutants were determined in parallel using the same batch of LB medium for growth. The relative mutation rate was then calculated by normalising all rates to the wild type determined in parallel in that particular experiment, hence setting the relative rate of wild type cells to 1.

## Spontaneous tandem repeat deletion rates

Plasmid pRS316-kankanMX4 was used for the determination of spontaneous tandem repeat deletion rates (see above). Measurements were carried out in analogy to the determination of spontaneous mutation rates the method of the median (Lea and Coulson 1949; Foster 2006; Ede et al. 2011). Appropriate dilutions from an overnight culture of the strain of interest were spread on LB agar plates to allow single cells to grow into colonies. 9 colonies were completely cut out of the agar with a sterile scalpel blade and transferred into a culture tube with 5 ml LB broth. The resulting 9 tubes were incubated for 24 h with agitation until bacterial growth reached stationary phase. Appropriate dilutions from all cultures were plated onto LB containing kanamycin (40 µg/ml) to determine the number of revertants in each culture. The viable titre was determined by plating appropriate dilutions of 3 randomly chosen cultures onto LB agar plates without antibiotic. The mean of the viable titres was used in the calculation of the mutation rate. For each set of experiments carried out at a certain time the mutation rate of wild type and mutants were determined in parallel using the same batch of LB medium for growth, as reversion rates can vary due to changes in media composition. The relative reversion rate was then calculated by normalising all rates to the wild type determined in parallel in that particular experiment, hence setting the relative rate of wild type cells to 1. All absolute rates are presented in Supplementary Table 2.

## Fluorescence Microscopy

Fluorescence microscopy was as described (Rudolph et al. 2007). Briefly, cells were grown to an *A_600_* of 0.2 in LB broth. A 1 ml sample was removed as unirradiated control. The rest of the cells were pelleted, UV-irradiated on the surface of LB agar and resuspended in the original, but filter-sterilised, supernatant to continue incubation. 1 ml samples were removed at the intervals indicated and transferred to a thin 1% LB agarose layer on microscopic slides. Cells were visualized with a BX-52 Olympus microscope equipped with a coolSNAP™HQ camera (Photometrics). YPet-DnaN foci were visualized using the JP4-CFP-YFP filterset 86002v2 (Chroma). Images were taken and analysed by MetaMorph 6.2 (Universal Imaging) and processed using MetaMorph and Adobe Photoshop CS6.

# Supplementary Tables

## Table S1. Effect of a recG deletion on the rate of spontaneous mutations in E. coli.

| Strain |  | spont. mutation rate [ × 10 ^–^ ^9^] | | relative spont.  mutation rate |
| --- | --- | --- | --- | --- |
| wild type | MG1655 | 3.52 | ± 0.71^a)^ | 1.00 |
| *recG* | N4560 | 1.95 | ± 0.46 ^a)^ | 0.60 |
| wild type | MG1655 | 4.10 | ± 0.63^b)^ | 1.00 |
| *recG* | N4560 | 2.60 | ± 0.43 ^b)^ | 0.63 |

Experiments wer performed two times independently (labelled a and b), producing very similar results. For all experiments the mutation rate of wild type and *recG* mutant was determined in parallel using the same batch of medium for growth. The relative mutation rate was calculated by normalising all rates to the wild type determined in parallel in the particular experiment.

## Table S2. Spontaneous tandem repeat deletion rates

| **Strain** |  | **Reversion Rate**  **[ × 10 ^–^ ^4^]** | | **Relative  Reversion Rate^a^** |
| --- | --- | --- | --- | --- |
| wild type | RCe014 | 5.29 | ± 0.34 | 1.00 |
| *recG* | RCe015 | 13.2 | ± 0.97 | 2.49 |
| *priA300 recG* | RCe030 | 22.2 | ± 1.47 | 4.19 |
| *recG priA300 ruvA60* | RCe047 | 13.4 | ± 0.93 | 2.53 |
| wild type | RCe014 | 13.5 | ± 0.87 | 1.00 |
| *recG recA* | RCe052 | 3.03 | ± 0.21 | 0.22 |
| wild type | RCe014 | 5.30 | ± 0.35 | 1.00 |
| *priA300* | RCe031 | 10.7 | ± 0.72 | 2.03 |
| wild type | RCe014 | 11.1 | ± 0.72 | 1.00 |
| *recA* | N4279 | 3.64 | ± 0.24 | 0.33 |
| wild type | RCe014 | 13.4 | ± 0.86 | 1.00 |
| *recG ruvA60* | RCe032 | 32.5 | ± 2.33 | 2.43 |
| wild type | RCe014 | 8.73 | ± 0.55 | 1.00 |
| *ruvA60* | RCe033 | 3.47 | ± 0.24 | 0.40 |
| wild type | RCe014 | 13.2 | ± 0.90 | 1.00 |
| *recB* | RCe045 | 3.88 | ± 0.33 | 0.29 |
| *recG recB* | RCe046 | 5.59 | ± 0.50 | 0.42 |
| wild type | RCe014 | 3.88 | ± 0.34 | 1.00 |
| *recF* | RCe125 | 2.26 | ± 0.18 | 0.60 |
| *recG recF* | RCe126 | 6.82 | ± 0.51 | 1.80 |

a – If absolute rates of mutant strains were determined on different days a wild type deletion rate was always run in parallel, as changes in media composition and culturing can influence absolute rates on different days. Relative rates were obtained by normalising the rate obtained to the rate of wild type cells determined in parallel. See Supplementary Methods for further details.

## Table S3. Spontaneous tandem repeat deletion rates

| **Strain** | **Relevant genotype** |  | **Conjugational crosses** | | | | | |  |
| --- | --- | --- | --- | --- | --- | --- | --- | --- | --- |
|  |  |  | No. Expts | Relative viability | Relative yield of transconjugants | | | | |
|  |  |  |  |  | × KL548 to Pro^+^ | × Hfr GY2200 | | × Hfr KL226 to Pro^+^ | |
|  |  |  |  |  |  | λ plaques | Thr^+^ Leu^+^ |  |  |
|  |  |  |  |  |  |  |  |  | |
| AB1157 | *ruv^+^ pri^+^ rec^+^* |  |  | **1.0 =** | **1.0 =** | **1.0 =** | **1.0 =** | **1.0 =** | |
|  | Mean |  | 43 | 1.57E+08 | 1.47E+07 | 4.39E+06 | 7.05E+06 | 8.75E+06 | |
|  | SE |  |  | 4.84E+06 | 6.63E+05 | 4.27E+05 | 3.22E+05 | 4.25E+05 | |
| AM2094 | *ΔruvABC recA* |  |  |  |  |  |  |  | |
|  |  |  | 3 | **0.65** | **0.60** | **0.64** | **0.000006** | **0.000015** | |
|  |  |  |  | 0.081 | 0.084 | 0.281 | 0.0000007 | 0.000005 | |
| AM2095 | *ΔruvABC recF* |  |  |  |  |  |  |  | |
|  |  |  | 4 | **0.65** | **0.81** | **0.91** | **0.78** | **0.84** | |
|  |  |  |  | 0.14 | 0.05 | 0.17 | 0.10 | 0.16 | |
| AM2123 | *ΔrecG* |  |  |  |  |  |  |  | |
|  |  |  | 4 | **0.82** | **0.70** | **0.89** | **0.35** | **0.25** | |
|  |  |  |  | 0.08 | 0.07 | 0.07 | 0.04 | 0.03 | |
| AM2124 | *ΔruvABC ΔrecG* |  |  |  |  |  |  |  | |
|  |  |  | 5 | **0.28** | **0.21** | **0.67** | **0.0018** | **0.0014** | |
|  |  |  |  | 0.07 | 0.03 | 0.10 | 0.00063 | 0.00025 | |
| AM2125 | *ΔruvABC ΔrecG recF143* |  |  |  |  |  |  |  | |
|  |  |  | 7 | **0.55** | **0.47** | **0.98** | **0.024** | **0.015** | |
|  |  |  |  | 0.05 | 0.02 | 0.05 | 0.003 | 0.002 | |
| AM2147 | *ΔruvABC ΔrecR* |  |  |  |  |  |  |  | |
|  |  |  | 3 | **0.85** | **0.72** | **0.77** | **0.58** | **0.45** | |
|  |  |  |  | 0.05 | 0.13 | 0.02 | 0.13 | 0.11 | |
| AM2148 | *ΔruvABC ΔrecO* |  |  |  |  |  |  |  | |
|  |  |  | 3 | **0.68** | **0.65** | **0.74** | **0.51** | **0.52** | |
|  |  |  |  | 0.06 | 0.18 | 0.15 | 0.09 | 0.05 | |
| AM2149 | *ΔruvABC recJ284* |  |  |  |  |  |  |  | |
|  |  |  | 3 | **0.62** | **0.82** | **1.09** | **0.49** | **0.75** | |
|  |  |  |  | 0.01 | 0.22 | 0.11 | 0.06 | 0.10 | |
| AM2160 | *ΔrecR* |  |  |  |  |  |  |  | |
|  |  |  | 3 | **1.04** | **0.98** | **0.94** | **0.85** | **0.64** | |
|  |  |  |  | 0.12 | 0.24 | 0.27 | 0.22 | 0.14 | |
| AM2161 | *ΔrecO* |  |  |  |  |  |  |  | |
|  |  |  | 3 | **0.94** | **0.89** | **0.87** | **0.71** | **0.66** | |
|  |  |  |  | 0.05 | 0.18 | 0.02 | 0.04 | 0.10 | |
| N2446 | *recJ284* |  |  |  |  |  |  |  | |
|  |  |  | 3 | **0.92** | **0.85** | **0.96** | **0.72** | **0.72** | |
|  |  |  |  | 0.12 | 0.22 | 0.07 | 0.01 | 0.07 | |
| N4361 | *recF143* |  |  |  |  |  |  |  | |
|  |  |  | 3 | **1.01** | **0.81** | **1.03** | **0.78** | **0.62** | |
|  |  |  |  | 0.11 | 0.10 | 0.17 | 0.02 | 0.06 | |
| N4454 | *ΔruvABC* |  |  |  |  |  |  |  | |
|  |  |  | 4 | **0.64** | **0.77** | **0.73** | **0.45** | **0.45** | |
|  |  |  |  | 0.06 | 0.13 | 0.12 | 0.06 | 0.05 | |
| N4549 | *ΔruvABC ΔrecG263::kan* |  |  |  |  |  |  |  | |
|  |  |  | 4 | **0.15** | **0.13** | **0.47** | **0.0009** | **0.0007** | |
|  |  |  |  | 0.03 | 0.01 | 0.11 | 0.0001 | 0.0001 | |
| N5501 | *priA300* |  |  |  |  |  |  |  | |
|  |  |  | 3 | **0.94** | **0.90** | **0.81** | **0.75** | **0.76** | |
|  |  |  |  | 0.12 | 0.11 | 0.06 | 0.02 | 0.13 | |
| N5510 | *priA300 ΔruvABC* |  |  |  |  |  |  |  | |
|  |  |  | 3 | **0.31** | **0.29** | **0.74** | **0.067** | **0.065** | |
|  |  |  |  | 0.06 | 0.01 | 0.04 | 0.01 | 0.001 | |
| N5512 | *priA300 ΔrecG* |  |  |  |  |  |  |  | |
|  |  |  | 3 | **0.86** | **0.89** | **0.80** | **0.76** | **0.81** | |
|  |  |  |  | 0.08 | 0.06 | 0.05 | 0.02 | 0.03 | |
| N5516 | *priA300 ruvABC recG263* |  |  |  |  |  |  |  | |
|  |  |  | 3 | **0.06** | **0.06** | **0.59** | **0.00041** | **0.0012** | |
|  |  |  |  | 0.01 | 0.01 | 0.20 | 0.0001 | 0.0002 | |
| N7079 | *ΔrecQ* |  |  |  |  |  |  |  | |
|  |  |  | 3 | **1.01** | **0.93** | **0.79** | **0.89** | **0.98** | |
|  |  |  |  | 0.16 | 0.12 | 0.12 | 0.10 | 0.09 | |
| N7082 | *ΔruvABC ΔrecQ* |  |  |  |  |  |  |  | |
|  |  |  | 3 | **0.67** | **0.68** | **0.87** | **0.48** | **0.57** | |
|  |  |  |  | 0.03 | 0.12 | 0.21 | 0.09 | 0.04 | |
| N7753 | *priA300 recF* |  |  |  |  |  |  |  | |
|  |  |  | 3 | **0.98** | **0.75** | **1.05** | **0.67** | **0.75** | |
|  |  |  |  | 0.11 | 0.01 | 0.11 | 0.05 | 0.18 | |
| N7755 | *priA300 recF recG ruvABC* |  |  |  |  |  |  |  | |
|  |  |  | 12 | **0.53** | **0.59** | **0.70** | **0.12** | **0.13** | |
|  |  |  |  | 0.03 | 0.03 | 0.04 | 0.01 | 0.01 | |
| N7759 | *priA300 recF recG263* |  |  |  |  |  |  |  | |
|  |  |  | 3 | **0.82** | **0.79** | **0.89** | **0.48** | **0.63** | |
|  |  |  |  | 0.08 | 0.05 | 0.06 | 0.01 | 0.12 | |
| N7774 | *priA300 recO recG ruvABC* |  |  |  |  |  |  |  | |
|  |  |  | 5 | **0.68** | **0.39** | **0.48** | **0.11** | **0.13** | |
|  |  |  |  | 0.05 | 0.03 | 0.11 | 0.02 | 0.01 | |
| N7775 | *priA300 recR recG ruvABC* |  |  |  |  |  |  |  | |
|  |  |  | 6 | **0.59** | **0.49** | **0.68** | **0.12** | **0.03** | |
|  |  |  |  | 0.04 | 0.10 | 0.08 | 0.02 | 0.004 | |
| N7780 | *priA300 recJ recG ruvABC* |  |  |  |  |  |  |  | |
|  |  |  | 3 | **0.28** | **0.35** | **0.28** | **0.02** | **0.02** | |
|  |  |  |  | 0.03 | 0.04 | 0.14 | 0.004 | 0.01 | |
| N7781 | *priA300 recQ recG ruvABC* |  |  |  |  |  |  |  | |
|  |  |  | 3 | **0.25** | **0.24** | **0.55** | **0.02** | **0.02** | |
|  |  |  |  | 0.07 | 0.01 | 0.08 | 0.001 | 0.0005 | |
| N7782 | *priA300 recF ruvABC* |  |  |  |  |  |  |  | |
|  |  |  | 3 | **0.54** | **0.91** | **0.61** | **0.53** | **0.51** | |
|  |  |  |  | 0.08 | 0.16 | 0.09 | 0.11 | 0.04 | |
| N7875* | *priA300 recG recO ruvABC recB* |  |  |  |  |  |  |  | |
|  |  |  | 3 | **0.45** | **0.43** | **1.12** | **0.00040** | **0.00043** | |
|  |  |  |  | 0.09 | 0.03 | 0.19 | 0.00003 | 0.000005 | |
| N7876* | *priA300 recG recR ruvABC recB* |  |  |  |  |  |  |  | |
|  |  |  | 3 | **0.48** | **0.47** | **1.00** | **0.00048** | **0.00024** | |
|  |  |  |  | 0.09 | 0.03 | 0.15 | 0.00003 | 0.00003 | |
| N7945 | *ΔruvABC rus-2* |  |  |  |  |  |  |  | |
|  |  |  | 3 | **0.84** | **1.02** | **1.07** | **1.04** | **1.01** | |
|  |  |  |  | 0.08 | 0.03 | 0.09 | 0.13 | 0.03 | |
| N7959 | *ΔruvABC rus-1* |  |  |  |  |  |  |  | |
|  |  |  | 3 | **0.93** | **1.18** | **1.42** | **1.04** | **1.10** | |
|  |  |  |  | 0.07 | 0.21 | 0.20 | 0.15 | 0.18 | |
| Mating in conjugational crosses was for 30 (KL548), 40 (KL226) or 60 (GY2200) min and the transconjugant class selected is as indicated. Values for wild type control strain AB1157 are set at 1, with the actual mean values over all experiments shown below ± standard errors (SE) as indicated. Mutant strains were tested in groups of 3–5 in parallel with AB1157 and the values shown in bold are mean yields relative to AB1157 in each of 3 or more independent experiments as indicated, with standard errors shown below the mean. ND = not determined.  *In these crosses, Hfr KL226 was replaced with Hfr KL227, which transfers in the same direction, but from a different point of F insertion such that, unlike KL226, it does not transfer the *recR* gene as an early marker. | | | | | | | | | |

## Table S4. Escherichia coli K-12 strains

| **Strain** | **Relevant Genotype^a^** | **Source^c^** |
| --- | --- | --- |
| **(a) AB1157 and W3110 derivatives^b^** | | |
| AB1157 | *araC14 thi-1 hisG4 Δ(gpt-proA)62 argE3 thr-1 leuB6 kdg51 rfbD1 araC14 lacY1 galK2 xyl-5 mtl-1 tsx-33 supE44 rac^–^  mgl-51 rpsL31qsr^–^* | (Bachmann, B J 1996) |
| W3110 | *F– λ– inv(rrnD–rrnE)1 rph-1* | (Bachmann, B J 1996) |
| AM1662 | *ΔrecO::dhfr* | (Mahdi et al. 2006) |
| AM1746 | *ΔrecO::kan* | A.A. Mahdi and R.G. Lloyd, unpublished |
| AM1816 | *ΔrecR::kan* | A.A. Mahdi and R.G. Lloyd, unpublished |
| AM2094 | *ΔruvABC::cat recA269::*Tn*10* | (Mahdi et al. 2012) |
| AM2095 | *ΔruvABC::cat tnaA::*Tn*10 recF143* | (Mahdi et al. 2012) |
| AM2123 | *ΔrecG::apra* | (Mahdi et al. 2012) |
| AM2124 | *ΔruvABC::cat ΔrecG::apra* | N4454 × P1.AM1655 to Apra^r^ |
| AM2125 | *ΔruvABC::cat tnaA::*Tn*10 recF143 ΔrecG::apra* | (Mahdi et al. 2012) |
| AM2147 | *ΔruvABC::cat ΔrecR::kan* | (Mahdi et al. 2012) |
| AM2148 | *ΔruvABC::cat ΔrecO::kan* | (Mahdi et al. 2012) |
| AM2149 | *ΔruvABC::cat recJ284::*Tn*10* | (Mahdi et al. 2012) |
| AM2160 | *ΔrecR::kan* | (Mahdi et al. 2012) |
| AM2161 | *ΔrecO::kan* | (Mahdi et al. 2012) |
| JC12334 | *tnaA::*Tn*10 recF143* | J.C. Clark |
| N2446 | *recJ284::*Tn*10* | (Mahdi et al. 2012) |
| N3005 | W3110 *purE85::*Tn*10* | (Lloyd and Buckman 1985) |
| N3793 | *ΔrecG263::kan* | (Mahdi et al. 1996) |
| N4361 | *tnaA::*Tn*10 recF143* | (Mahdi et al. 2012) |
| N4454 | *ΔruvABC::cat* | (Jaktaji and Lloyd 2003) |
| N4549 | *ΔruvABC::cat ΔrecG263::kan* | N4454 × P1.N3793 to Km^r^ |
| N4574 | *relA1 ΔspoT207::cat rpoB*35 ΔruvC65 eda-51::*Tn*10  rus-2 (orf-56::IS10)* | (Mahdi et al. 2006) |
| N4884 | *rpoB*35 ΔruvABC::cat* | (Mahdi et al. 2006) |
| N5501 | *argE^+^ priA300* | (Jaktaji and Lloyd 2003) |
| N5510 | *argE^+^ priA300 ΔruvABC::cat* | N5501 × P1.N4454 to Cm^r^ |
| N5512 | *argE^+^ priA300 ΔrecG263::kan* | N5501 × P1.N3793 to Km^r^ |
| N5516 | *argE^+^ priA300 ΔruvABC::cat ΔrecG263::kan* | N5510 × P1.N3793 to Km^r^ |
| N5602 | *ΔrecQ::kan* | (Mahdi et al. 2006) |

| N6822 | *ΔlacIZYA Δrep::cat dnaC809,820 zji-202::*Tn*10 ΔpriA::apra recR1::EZdhfr* | R.G. Lloyd, unpublished |
| --- | --- | --- |
| N7079 | *ΔrecQ::kan* | (Zhang et al. 2010) |
| N7082 | *ΔruvABC::cat ΔrecQ::kan* | (Zhang et al. 2010) |
| N7753 | *argE^+^ priA300 tnaA::*Tn*10 recF143* | N5501 × P1.JC12334 to Tc^r^ |
| N7754 | *argE^+^ priA300 ΔrecG263::kan tnaA::*Tn*10 recF143* | N5512 × P1.JC12334 to Tc^r^ |
| N7755 | *argE^+^ priA300 ΔrecG263::kan tnaA::*Tn*10 recF143 ΔruvABC::cat* | N7754 × P1.N4884 to Cm^r^ |
| N7759 | *argE^+^ priA300 tnaA::*Tn*10 recF143 ΔrecG263::kan* | N7753 × P1.N3793 to Km^r^ |
| N7772 | *argE^+^ priA300 ΔrecG263::kan ΔrecO::dhfr* | N5512 × P1.AM1662 to Tm^r^ |
| N7773 | *argE^+^ priA300 ΔrecG263::kan recR1::EZdhfr* | N5512 × P1.N6822 to Tm^r^ |
| N7774 | *argE^+^ priA300 ΔrecG263::kan ΔrecO::dhfr ΔruvABC::cat* | N7772 × P1.N4884 to Cm^r^ |
| N7775 | *argE^+^ priA300 ΔrecG263::kan recR1::EZdhfr ΔruvABC::cat* | N7773 × P1.N4884 to Cm^r^ |
| N7777 | *argE^+^ priA300 ΔrecG::apra* | N5512 × P1.AM2123 to Apra^r^ Km^s^ |
| N7778 | *argE^+^ priA300 ΔrecG263::kan recJ284::*Tn*10* | N5512 × P1.N4934 to Tc^r^ |
| N7779 | *argE^+^ priA300 ΔrecG::apra ΔrecQ::kan* | N7777 × P1.N5602 to Km^r^ |
| N7780 | *argE^+^ priA300 ΔrecG263::kan recJ284::*Tn*10 ΔruvABC::cat* | N7778 × P1.N4884 to Cm^r^ |
| N7781 | *argE^+^ priA300 ΔrecG::apra ΔrecQ::kan ΔruvABC::cat* | N7779 × P1.N4884 to Cm^r^ |
| N7782 | *argE^+^ priA300 tnaA::*Tn*10 recF143 ΔruvABC::cat* | N7753 × P1.N4884 to Cm^r^ |
| N7875 | *argE^+^ priA300 ΔrecG263::kan ΔrecO::dhfr ΔruvABC::cat recB268::*Tn*10* | N7774 × P1.TRM308 to Tc^r^ |
| N7876 | *argE^+^ priA300 ΔrecG263::kan recR1::dhfr ΔruvABC::cat recB268::*Tn*10* | N7775 × P1.TRM308 to Tc^r^ |
| N7944 | *ΔruvABC::cat purE85::*Tn*10* | N4454 × P1.N3005 to Tc^r^ |
| N7945 | *ΔruvABC::cat rus-2 (orf-56::IS10)* | N7944 × P1.N4574 to *purE^+^* |
| N7959 | *ΔruvABC::cat rus-1 (orf-151::IS2)* | R.G. Lloyd, unpublished |
| RRL190 | *kan-ypet-dnaN* | David Sherratt |
| TRM308 | *ΔargE::I-SceIcs-cat recB268::*Tn*10 sbcA* | (Mahdi et al. 2006) |

| **(b) MG1655 derivatives^b^** | | |
| --- | --- | --- |
| MG1655 | F– *rph-1* | (Bachmann, B J 1996) |
| AM1655 | *ΔrecG::apra* | (Mahdi et al. 2006) |
| AS1062 | *kan-ypet-dnaN* | MG1655 × P1.RRL190 to Km^r^ |
| AS1103 | *ΔlacIZYA ΔsbcCD::spc ΔxseA::dhfr ΔxonA::apra* pAM401 pAST116 | N7684 × pAST116 to Km^r^ |
| JD1099 | *ΔxseA::dhfr ΔsbcCD::kan ΔxonA::apra* | RCe570 × P1.AS1103 to Apra^r^ |
| N4239 | *ruvA60::*Tn*10* | (Meddows et al. 2004) |
| N4278 | *recB268::*Tn*10* | (Meddows et al. 2004) |
| N4279 | *recA269::*Tn*10* | (Meddows et al. 2004) |
| N4560 | *recG265::cat* | (Mahdi et al. 2006) |
| N4934 | *recJ284::*Tn*10* | (Rudolph et al. 2008) |
| N5189 | *ruvA60::*Tn*10 recG265::cat* | R.G. Lloyd, unpublished |
| N5296 | *xonAΔ300::cat ΔsbcCD::kan* | R.G. Lloyd, unpublished |
| N5500 | *priA300* | (Jaktaji and Lloyd 2003) |
| N5511 | *priA300 recG265::cat* | (Rudolph et al. 2009) |
| N5540 | *tnaA::*Tn*10 recF143* | (Rudolph et al. 2008) |
| N7684 | *ΔlacIZYA ΔsbcCD::spc ΔxseA::dhfr ΔxonA::apra* pAM401 | (Rudolph et al. 2010) |
| RCe014 | pRS316-kankanMX4 | MG1655 × pRS316-kankanMX4 to Ap^r^ |
| RCe015 | *recG265::cat* pRS316-kankanMX4 | N4560 × pRS316-kankanMX4 to Ap^r^ |
| RCe027 | *recG265::cat recA269::*Tn*10* | N4560 × P1.N4279 to Tc^r^ |
| RCe030 | *priA300 recG265::cat* pRS316-kankanMX4 | N5511 × pRS316-kankanMX4 to Ap^r^ |
| RCe031 | *priA300* pRS316-kankanMX4 | N5500 × pRS316-kankanMX4 to Ap^r^ |
| RCe032 | *ruvA60::*Tn*10 recG265::cat* pRS316-kankanMX4 | N5189 × pRS316-kankanMX4 to Ap^r^ |
| RCe033 | *ruvA60::*Tn*10* pRS316-kankanMX4 | N4239 × pRS316-kankanMX4 to Ap^r^ |
| RCe040 | *recB268::*Tn*10 recG265::cat* | N4560 × P1.N4278 to Tc^r^ |
| RCe041 | *priA300 recG265::cat ruvA60::*Tn*10* | N5511 × P1.N4239 to Tc^r^ |
| RCe045 | *recB268::*Tn*10* pRS316-kankanMX4 | N4278 × pRS316-kankanMX4 to Ap^r^ |
| RCe046 | *recB268::*Tn*10 recG265::cat* pRS316-kankanMX4 | RCe040 × pRS316-kankanMX4 to Ap^r^ |
| RCe047 | *priA300 recG265::cat ruvA60::*Tn*10* pRS316-kankanMX4 | RCe041 × pRS316-kankanMX4 to Ap^r^ |
| RCe052 | *recG265::cat recA269::*Tn*10* pRS316-kankanMX4 | RCe027 × pRS316-kankanMX4 to Ap^r^ |
| RCe121 | *tnaA::*Tn*10 recF143 recG265::cat* | N5540 × P1.N4560 to Cm^r^ |
| RCe125 | *tnaA::*Tn*10 recF143* pRS316-kankanMX4 | N5540 × pRS316-kankanMX4 to Ap^r^ |
| RCe126 | *tnaA::*Tn*10 recF143 recG265::cat* pRS316-kankanMX4 | RCe121 × pRS316-kankanMX4 to Ap^r^ |
| RCe562 | *ΔsbcCD::kan* | MG1655 × P1.N5296 to Km^r^ |
| RCe563 | *ΔxonA::apra* | MG1655 × P1.AS1103 to Apra^r^ |
| RCe564 | *ΔxseA::dhfr* | MG1655 × P1.AS1103 to Tm^r^ |
| RCe568 | *ΔxonA::apra ΔxseA::dhfr* | RCe563 × P1.AS1103 to Tm^r^ |
| RCe569 | *ΔxonA::apra ΔsbcCD::kan* | RCe563 × P1.N5296 to Km^r^ |
| RCe570 | *ΔxseA::dhfr ΔsbcCD::kan* | RCe564 × P1.N5296 to Km^r^ |
| **(c) Hfr and F-prime donors** | | |
| GY2200 | Hfr (H, PO1) (λ*ind*)^+^ *thi-1 relA1* | R. Devoret |
| KL226 | Hfr (Cavalli, PO2A) *relA1 tona22* | K. B. Low |
| KL227 | Hfr (PO3 of P4X) *metB1* | K. B. Low |
| KL548 | F’ (F128) *lacI3 lacZ118 proAB^+^* | K. B. Llow |

a – The abbreviations *kan*, *cat*, *dhfr* and *apra* refer to insertions conferring resistance to kanamycin (Km^r^), chloramphenicol (Cm^r^), trimethoprim (Tm^r^) and apramycin (Apra^r^), respectively. Tn*10* confers resistance to tetracycline (Tc^r^). Plasmids carry an ampicillin (Amp^r^) resistance marker. For all relevant plasmid details see Supplementary Methods.

b – Only the relevant additional genotype of the derivatives is shown.

# Supplementary References

Bachmann, B J (1996) Derivations and Genotypes of Some Mutant Derivatives of *Escherichia coli* K-12. In: *Escherichia coli* and Salmonella Cellular and Molecular Biology, Second Edition. ASM Press,

Datsenko KA, Wanner BL (2000) One-step inactivation of chromosomal genes in *Escherichia* *coli* K-12 using PCR products. Proc Natl Acad Sci U S A 97:6640–6645. doi: 10.1073/pnas.120163297

Dimude JU, Stockum A, Midgley-Smith SL, et al (2015) The Consequences of Replicating in the Wrong Orientation: Bacterial Chromosome Duplication without an Active Replication Origin. mBio. doi: 10.1128/mBio.01294-15

Ede C, Rudolph CJ, Lehmann S, et al (2011) Budding yeast Mph1 promotes sister chromatid interactions by a mechanism involving strand invasion. DNA Repair 10:45–55. doi: 10.1016/j.dnarep.2010.09.009

Foster PL (2006) Methods for determining spontaneous mutation rates. Methods Enzymol 409:195–213. doi: 10.1016/S0076-6879(05)09012-9

Jaktaji RP, Lloyd RG (2003) PriA supports two distinct pathways for replication restart in UV-irradiated *Escherichia* *coli* cells. Mol Microbiol 47:1091–1100.

Lea DE, Coulson CA (1949) The distribution of the numbers of mutants in bacterial populations. J Genet 49:264–285.

Lloyd RG, Buckman C (1985) Identification and genetic analysis of *sbcC* mutations in commonly used *recBC* *sbcB* strains of *Escherichia* *coli* K-12. J Bacteriol 164:836–844.

Lloyd RG, Evans NP, Buckman C (1987) Formation of recombinant *lacZ*^+^ DNA in conjugational crosses with a *recB* mutant of *Escherichia* *coli* K12 depends on *recF*, *recJ*, and *recO*. Mol Gen Genet MGG 209:135–141.

Lloyd RG, Porton MC, Buckman C (1988) Effect of *recF, recJ, recN, recO* and *ruv* mutations on ultraviolet survival and genetic recombination in a *recD* strain of *Escherichia* *coli* K12. Mol Gen Genet MGG 212:317–324.

Mahdi AA, Briggs GS, Lloyd RG (2012) Modulation of DNA damage tolerance in *Escherichia coli recG* and *ruv* strains by mutations affecting PriB, the ribosome and RNA polymerase. Mol Microbiol 86:675–691. doi: 10.1111/mmi.12010

Mahdi AA, Buckman C, Harris L, Lloyd RG (2006) Rep and PriA helicase activities prevent RecA from provoking unnecessary recombination during replication fork repair. Genes Dev 20:2135–2147. doi: 10.1101/gad.382306

Mahdi AA, Sharples GJ, Mandal TN, Lloyd RG (1996) Holliday junction resolvases encoded by homologous *rusA* genes in *Escherichia coli* K-12 and phage 82. J Mol Biol 257:561–573. doi: 10.1006/jmbi.1996.0185

Meddows TR, Savory AP, Lloyd RG (2004) RecG helicase promotes DNA double-strand break repair. Mol Microbiol 52:119–132. doi: 10.1111/j.1365-2958.2003.03970.x

Rudolph CJ, Mahdi AA, Upton AL, Lloyd RG (2010) RecG protein and single-strand DNA exonucleases avoid cell lethality associated with PriA helicase activity in *Escherichia coli*. Genetics 186:473–492. doi: 10.1534/genetics.110.120691

Rudolph CJ, Upton AL, Harris L, Lloyd RG (2009) Pathological replication in cells lacking RecG DNA translocase. Mol Microbiol 73:352–366. doi: 10.1111/j.1365-2958.2009.06773.x

Rudolph CJ, Upton AL, Lloyd RG (2007) Replication fork stalling and cell cycle arrest in UV-irradiated *Escherichia coli*. Genes Dev 21:668–681. doi: 10.1101/gad.417607

Rudolph CJ, Upton AL, Lloyd RG (2008) Maintaining replication fork integrity in UV-irradiated *Escherichia coli* cells. DNA Repair 7:1589–1602. doi: 10.1016/j.dnarep.2008.06.012

Sikorski RS, Hieter P (1989) A system of shuttle vectors and yeast host strains designed for efficient manipulation of DNA in *Saccharomyces cerevisiae*. Genetics 122:19–27.

Thomason LC, Costantino N, Court DL (2007) *E. coli* genome manipulation by P1 transduction. Curr Protoc Mol Biol Ed Frederick M Ausubel Al Chapter 1:Unit 1.17. doi: 10.1002/0471142727.mb0117s79

Wach A, Brachat A, Pöhlmann R, Philippsen P (1994) New heterologous modules for classical or PCR-based gene disruptions in *Saccharomyces cerevisiae*. Yeast Chichester Engl 10:1793–1808.

Zhang J, Mahdi AA, Briggs GS, Lloyd RG (2010) Promoting and avoiding recombination: contrasting activities of the *Escherichia coli* RuvABC Holliday junction resolvase and RecG DNA translocase. Genetics 185:23–37. doi: 10.1534/genetics.110.114413
